# Priming and elongation of chitin chains: Implications for chitin synthase mechanism

**DOI:** 10.1016/j.tcsw.2018.100017

**Published:** 2018-12-30

**Authors:** Peter Orlean, Danielle Funai

**Affiliations:** Department of Microbiology, University of Illinois at Urbana-Champaign, Urbana, IL 61801, USA

**Keywords:** Chitin synthesis, Fungal cell wall, Polysaccharide

## Abstract

Most fungi have multiple chitin synthases (CSs) that may make chitin at different sites on the cell surface, at different times during growth, and in response to cell wall stress. The structure-based model for CS function is for transfer of GlcNAc from UDP-GlcNAc at the cytoplasmic face of the plasma membrane to the non-reducing end of a growing chitin chain, which is concomitantly translocated through a transmembrane channel formed by the synthase. Two aspects of CS mechanism are investigated: how chains might be initiated, and what governs how long they can get. First, it is shown that CSs incorporate free GlcNAc into di-*N*-acetylchitobiose and into insoluble chitin in a UDP-GlcNAc-dependent manner, and therefore that GlcNAc primes chitin synthesis. Second, average lengths of insoluble chitin chains were measured by determining the molar ratio of priming GlcNAc to chain-extending, UDP-GlcNAc-derived GlcNAc, and showed dependence on UDP-GlcNAc concentration, approaching a maximum at higher concentrations of substrate. These results, together with previous findings that 2-acylamido GlcN analogues prime formation of chitin oligosaccharides and stimulate chitin synthesis are discussed in the context of the structure-based model, and lead to the following proposals. 1) CSs may “self-prime” by hydrolyzing UDP-GlcNAc to yield GlcNAc. 2) A CS’s active site is not continuously occupied by a nascent chitin chain, rather, CSs can release chitin chains, then re-initiate, and therefore synthesize chitin chains in bursts. 3) The length of chitin chains made by a given CS will impact that CS’s contribution to construction of the fungal cell wall.

## Introduction

Chitin, a linear polymer of β1,4-linked *N*-acetylglucosamine, is a signature component of the walls of many fungi. This polysaccharide has a structural role because extensive hydrogen bonding between individual chains renders it insoluble, and because chitin chains can be cross-linked to other cell wall polymers (reviewed by Orlean, 2012, Cabib and Arroyo, 2013, Roncero et al., 2016).

Many fungal species have multiple chitin synthase genes. Studies of fungi lacking one or more of their chitin synthase genes suggest that the chitin in their cell walls is likely to be the product of two or more chitin synthases (CSs)^1^ that are active at different sites in the plasma membrane and at different times during cell growth and division (Shaw et al., 1991, Muszkieta et al., 2014, Lenardon et al., 2007, Roncero et al., 2016). CSs are members of Family 2 of inverting glycosyltransferases (GT-2) (Cantarel et al., 2009), which includes cellulose synthases. The current model for the mechanism of GT-2 Family processive polysaccharide synthases, which is inferred from the structure of the bacterial cellulose synthase BcsA (Morgan et al., 2013), is for transfer of single sugars from a donor sugar nucleotide to the non-reducing end of a growing glycan chain. This proceeds via an S_N_2-like displacement mechanism in which the C4 hydroxyl group at the non-reducing end of the acceptor glycan attacks the anomeric C1 of the sugar of the donor UDP-linked sugar, with release of UDP as leaving group (Lairson et al., 2008, McNamara et al., 2015). The nascent glycan chain is concomitantly extruded through the membrane via a pore made up of transmembrane helices of the synthase. The BcsA structure has been used to model an analogous, non-reducing end extension mechanism for the yeast CS, Chs2 (Dorfmueller et al., 2014).

The model for the mechanism of GT Family 2 polysaccharide synthases depicts transfer of monosaccharide from a sugar nucleotide to a preexisting chain in a synthase’s translocation channel, but it remains open how a glycan chain is initiated *de novo*. Potential initiation steps include a reaction between two UDP-linked saccharides to generate a UDP-linked disaccharide, or monosaccharide transfer from a UDP-sugar to some low molecular weight primer or intermediate. Formation of a UDP-linked disaccharide, the expected initiation product of a reducing end extension mechanism, is inconsistent with the structure-based model for non-reducing end chain elongation (Morgan et al., 2013).

There is so far no indication that lipid-linked intermediates are involved in chitin synthesis. First, the known GlcNAc-containing lipids formed in yeast – the dolichol pyrophosphate linked precursors in N-glycosylation and the glycosylphosphatidylinositol (GPI) precursor glycolipids – are not chitin precursors, and indeed, defects in N-glycosylation and GPI anchoring are associated with increased chitin synthesis (reviewed by Orlean, 2012). Moreover, tunicamycin, an inhibitor of N-linked glycosylation (Lehle and Tanner, 1976) had no effect on the *in vitro* synthesis of chitin by *S. cerevisiae* CSs (Duran and Cabib, 1977, Orlean, 1987. Second, no potential glycolipid-intermediates in chitin synthesis have been recovered from *in vitro* chitin synthase incubations (Duran and Cabib, 1977) or detected in studies of the N-linked glycan- and GPI anchor precursor glycolipids that are formed when yeast membranes are incubated with UDP-GlcNAc (Lehle and Tanner, 1976, Costello and Orlean, 1992, Leidich et al., 1994). Third, no genetic screen for chitin synthesis-related genes in yeast has so far uncovered clues for any additional chitin biosynthetic step between UDP-GlcNAc and chitin (Roncero, 2002, Lesage et al., 2005). Fourth, it was concluded from the BcsA structure that a membrane-associated, lipid-linked Glc donor would not reach the enzyme’s catalytic site (Morgan et al., 2013), and the same distance constraints may well apply for CSs.

**Fig. 1:**
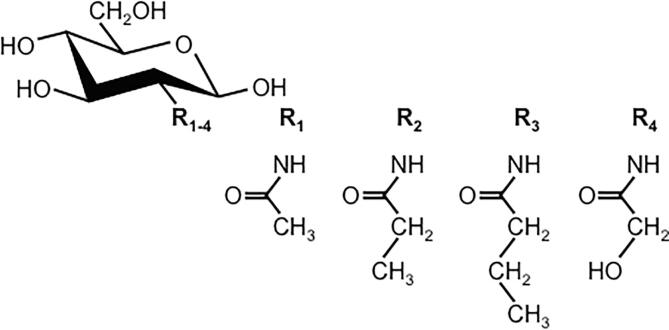
Structures of GlcNAc and 2-acylamido GlcN derivatives. Substituents at the 2-position, denoted by R, are as follows. R_1_: N-acetyl, R_2_: N-propionyl, R_3_: N-butanoyl, R_4_: N-glycolyl (Gyore et al., 2014).

Free GlcNAc- or chitin-oligosaccharides (COs)^2^ remain candidates for primers of chitin synthesis. *In vitro* activity of CSs in yeast membranes can be stimulated few- to many fold when free GlcNAc is included in incubations (Keller and Cabib, 1971, McMurrough and Bartnicki-Garcia, 1971, Fähnrich and Ahlers, 1981, Sburlati and Cabib, 1986, Cabib, 1987, Orlean, 1987, Horsch et al., 1996), and GlcNAc has been suggested to serve as a co-substrate (Fähnrich and Ahlers, 1981) or an allosteric activator (Porter and Jaworski, 1966, Camargo et al., 1967, Horsch et al., 1996) in the CS reaction. The findings with yeast Chs2 that 2-acylamido analogues of GlcN (Fig. 1) are acceptors for GlcNAc transfer from UDP-GlcNAc, then extended into chitin oligosaccharides by further transfers from UDP-GlcNAc, and that these analogues also stimulate insoluble chitin synthesis, strongly suggest that GlcNAc itself can prime CO and chitin formation *in vitro* (Gyore et al., 2014).

GT Family 2 processive synthases presumably make a distribution of chain lengths. For CSs, the few estimates reported are of average chain lengths of 50–200 GlcNAcs (Kang et al., 1984, Orlean, 1987, Grabinska et al., 2007). Different CSs may inherently make longer or shorter chain length distributions at a fixed UDP-GlcNAc concentration, and, because the dependence of yeast CS activity on UDP-GlcNAc concentration can be described by Michaelis-Menten kinetics (Keller and Cabib, 1971, Kang et al., 1984, Sburlati and Cabib, 1986, Orlean, 1987), the rate of monomer addition to chitin chains will depend on UDP-GlcNAc concentration at low concentrations of this substrate, leading in turn to the prediction that shorter chains will be generated at lower UDP-GlcNAc concentrations. The size of a CS’s product may impact the contribution that CS will make to cell wall biogenesis.

Here, we report that free GlcNAc primes formation of di-*N*-acetylchitobiose and insoluble chitin by a yeast CS. Based these findings, we devised a strategy for estimating average chain length of insoluble chitin by determining the ratio of priming GlcNAc to chain-extending, UDP-derived GlcNAc in product, and we used this approach to show that chitin chain length increases with UDP-GlcNAc concentration at low concentrations of this sugar nucleotide donor.

These results, together with previous findings that 2-acylamido GlcN analogues prime CO synthesis and stimulate formation of insoluble chitin (Gyore et al., 2014) are discussed in the context of the structure-based model for processive GT-2 Family enzymes.

## Materials and methods

### Yeast strains, culture conditions, and membrane isolation

*S. cerevisiae* strains YEF7489 (*chs1*Δ *chs3*Δ), YEF7492 (*chs1*Δ *chs3*Δ harboring pRS314 (CEN, *TRP1*), and YEF7493 (*chs1*Δ *chs3*Δ harboring pYO201 (*CHS2* overexpresser)) had the genotypes described previously (Gyore et al., 2014) were obtained from Y. Oh and E. Bi (University of Pennsylvania Philadelphia, USA). Plasmid pBG1805-*CHS1* was obtained from Dharmacon, Inc., Lafayette, CO, USA and introduced into strain YEF7489. Chs1 and Chs2 expression were induced in medium containing galactose and raffinose, and mixed membranes were prepared essentially as described by Oh and coworkers (Oh et al., 2012), except that the raffinose concentration used was 2% (w/v), and that glycerol was left out of the final buffer that membranes were frozen in (Gyore et al., 2014). Prior to assay, portions of mixed membranes containing overexpressed Chs1 were pre-treated with trypsin (200 μg/mL final concentration) for 10 min at 30 °C, after which trypsin inhibitor (400 μg/mL final concentration) was added (Orlean, 1987, Oh et al., 2012).

### Standard assay for CO formation

Incubations with UDP-[^14^C]GlcNAc and 32 mM unlabeled GlcNAc, isolation of Dowex 1 × 8 column run-throughs containing COs, and thin layer chromatography (TLC) were carried out as described (Gyore et al., 2014), except that for assays of Chs1, incubations contained 5 mM MgCl_2_ instead of cobalt acetate.

### [^14^C]GlcNAc labeling of COs and insoluble chitin: [^14^C]GlcNAc priming assay

Mixed membranes from a control *chs1*Δ *chs3*Δ strain harboring an empty vector and incubated in parallel in galactose and raffinose, or from *chs1*Δ *chs3*Δ strains overexpressing *Chs1* or *Chs2* were assayed. Incubations were carried out in a final volume of 50 μL in 30 mM Tris-HCl buffer pH 7.5 containing 2 μCi [^14^C]GlcNAc (sp. act.: 55 mCi/mmol, American Radiolabeled Chemicals, St. Louis, MO, USA), which gave a final concentration of 0.72 mM GlcNAc. When included, unlabeled UDP-GlcNAc was present at a final concentration of 2 mM. In some [^14^C]GlcNAc priming assays, additional unlabeled GlcNAc was added from stock solutions to give final concentrations of 3.9 or 16.7 mM. In some experiments, 2 mM UDP-GlcNAc_2_ or 2 mM 4-O-methyl GlcNAc replaced UDP-GlcNAc. Incubations were for 45 min at 30 °C. Dowex 1 × 8 column run-throughs obtained as in the standard CO formation protocol were next applied to 3 mL bed volume charcoal-Celite columns poured in disposable plastic 10 mL pipets, and columns eluted and [^14^C]GlcNAc-depleted CO-fractions obtained as detailed by Gyore and coworkers (Gyore et al., 2014). Separation of the CO fractions by thin layer chromatography (TLC)^3^ was performed as described (Gyore et al., 2014). In some experiments, the insoluble material remaining after chloroform/methanol/water extraction, phase partitioning, and collection of the CO-containing fraction was precipitated in 10% (w/v) trichloroacetic acid after removal of the organic phase, the precipitate collected on glass fiber filters, and its radioactivity measured by scintillation counting.

### Determination of ratios of priming GlcNAc to chain-extending, UDP-derived GlcNAc

Mixed membranes from control and *Chs2*-overexpressing strains were assayed in parallel. Conditions for [^14^C]GlcNAc labeling were the same as those used in the [^14^C]GlcNAc priming protocol, except that the final concentration of GlcNAc was kept constant at 2.18 mM, and final concentrations of unlabeled UDP-GlcNAc were 0.1, 0.3, 1.0, and 3.0 mM. For measurement of UDP-GlcNAc-derived, chain extending GlcNAc, incubation mixtures contained 0.05 μCi UDP-[^14^C]GlcNAc and 2.18 mM unlabeled GlcNAc. Final concentrations of UDP-GlcNAc were adjusted to 0.1, 0.3, 1.0, and 3.0 mM from stock solutions of unlabeled GlcNAc. Incubations were for 30 min at 30 °C. Following extraction of COs according to the standard protocol, and removal of the organic phase remaining after solvent extraction, 1 mg of finely ground carrier glycogen was added as a suspension in 0.38 mL chloroform/methanol (3:2 v/v). Insoluble chitin and glycogen were dispersed by sonication, centrifuged for 10 min in a microfuge, and the supernatant discarded. The pellet was then washed twice more with 0.38 mL of chloroform/methanol (3:2 v/v), twice with chloroform/methanol/water (10:10:3 v/v/v), and three times with 50% aqueous ethanol. Insoluble material was then washed into a vial for scintillation counting. The radioactivity remaining in insoluble material after incubations with control membranes was taken to represent background, and was subtracted from the radioactivity measured in corresponding incubations with *CHS2*-overexpressing membranes.

### Reagents

[^14^C]GlcNAc (sp. act.: 55 mCi/mmol) and UDP-[^14^C]GlcNAc (sp. act. 300 mCi/mmol) were obtained from American Radiolabeled Chemicals, St. Louis, MO, USA), and UDP-GlcNAc_2_ and UDP-4-O-methyl GlcNAc were custom synthesized by the Peptide Institute, Osaka, Japan). UDP-GlcNAc and glycogen were from Sigma.

## Results

### Free GlcNAc is incorporated into GlcNAc_2_ and insoluble chitin in a UDP-GlcNAc-dependent manner

Our findings that yeast Chs2 transferred GlcNAc from UDP-GlcNAc to 2-acylamido analogues of glucosamine, and that the resulting disaccharides were extended by transfer of further GlcNAcs (Gyore et al., 2014) were a strong indication that free GlcNAc itself can prime CO formation. We tested this, as well as the possibility that free GlcNAc is incorporated into insoluble chitin, by performing CS assays in which [^14^C]GlcNAc was used as potential primer, in the presence or absence of unlabeled UDP-GlcNAc, using yeast membranes overexpressing Chs2 as well as control membranes. CO fractions were isolated as described in Section “[^14^C]GlcNAc labeling of COs and insoluble chitin: [^14^C]GlcNAc priming assay”, and submitted to TLC.

**Fig. 2:**
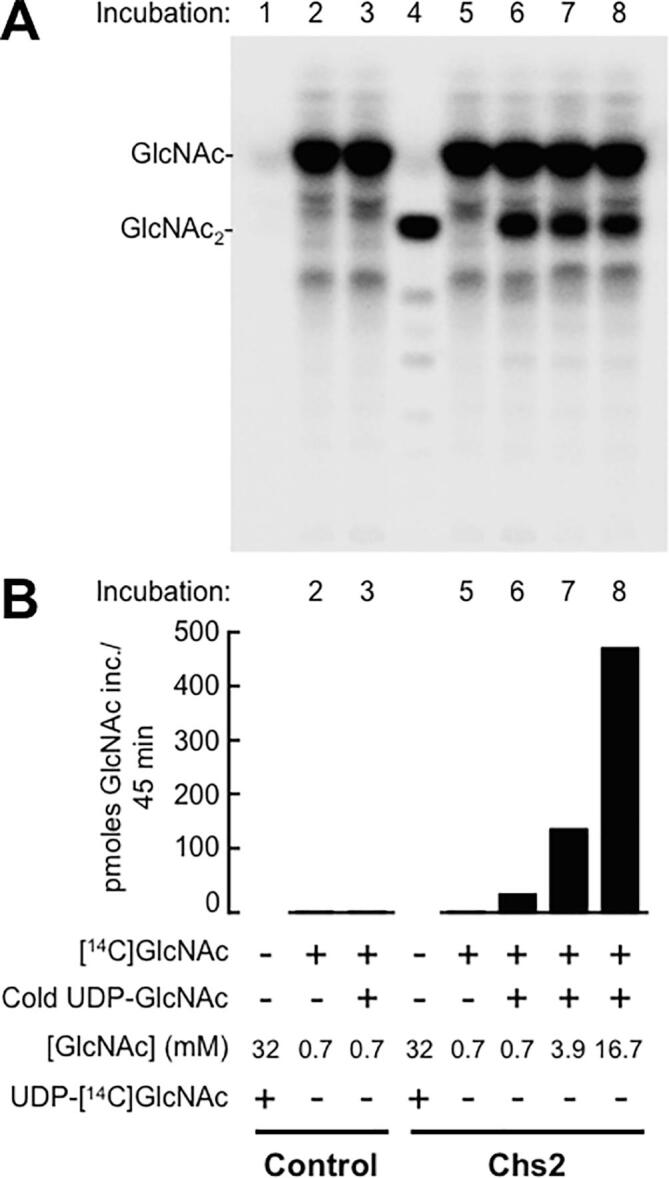
Priming of chitin oligosaccharide and insoluble chitin synthesis by [^14^C]GlcNAc. A. Incorporation of [^14^C]GlcNAc into GlcNAc_2_ in the presence of unlabeled UDP-GlcNAc and in the presence of increasing concentrations of GlcNAc. COs were separated by TLC and detected by phosphorimaging. Lanes 1–3 contain CO fractions from incubations with control membranes, and lanes 4–8, CO fractions from incubations with membranes overexpressing yeast Chs2. Incubations 1 and 4 were with UDP-[^14^C]GlcNAc and 32 mM unlabeled GlcNAc and confirm Chs2-overexpression-dependent formation of COs (lane 4). Lanes 5–8 display CO fractions from incubations with [^14^C]GlcNAc and the indicated final concentrations of GlcNAc, and either no UDP-GlcNAc (lane 5) or 2 mM unlabeled UDP-GlcNAc (lanes 6–8). CO fractions in lanes 2, 3, and 5–8 were submitted to charcoal-Celite chromatography to remove excess [^14^C]GlcNAc. B. Incorporation of [^14^C]GlcNAc into insoluble chitin made in the absence (incubations 2 and 5) or presence (incubations 6–8) of unlabeled UDP-GlcNAc, and in the presence of increasing concentrations of GlcNAc (incubations 6–8). Incubations 2 and 3 were with control membranes from yeast expressing only chromosomally encoded Chs2, and incubations 5–8 were with membranes overexpressing Chs2. The insoluble material quantified in Panel B’s incubations 2, 3, 5–8 was formed in the correspondingly numbered incubations in panel A.

In the presence of unlabeled UDP-GlcNAc, membranes containing overexpressed Chs2 formed a radiolabeled product with the same thin layer chromatographic mobility as GlcNAc_2_ (Fig. 2A, incubations 6–8), whereas no such product was formed by control membranes (Fig. 2A, incubation 3). In the absence of unlabeled UDP-GlcNAc, no [^14^C]GlcNAc_2_ was formed (Fig. 2A, incubations 2 and 5). In incubations with Chs2-overexpressing membranes, but not with membranes from control cells, [^14^C]GlcNAc was also incorporated into insoluble material when unlabeled UDP-GlcNAc was present (Fig. 2B, incubations 6–8), but not when unlabeled UDP-GlcNAc was omitted (Fig. 2B, incubation 5). Exochitinase treatment of this radiolabeled, insoluble material released [^14^C]GlcNAc (not shown). The simplest explanation for these results is that Chs2 catalyzed the displacement of UDP from UDP-GlcNAc by [^14^C]GlcNAc to form [^14^C]-labeled di-N-acetylchitobiose, which was subsequently extended into insoluble chitin by further transfers of GlcNAc from UDP-GlcNAc, leaving the single priming GlcNAc at one end of the chitin chain. Importantly, because neither GlcNAc_2_ nor insoluble chitin were formed in incubations with [^14^C]GlcNAc in the absence of unlabeled UDP-GlcNAc, we can conclude that Chs2 does not catalyze the joining of two or more free GlcNAc residues.

As the concentration of GlcNAc was increased up to 24-fold, formation of [^14^C]GlcNAc_2_ was still detected (Fig. 2A, incubations 6–8). Incorporation of GlcNAc into insoluble material could be quantified by correcting for specific activity of [^14^C]GlcNAc in incubations, and this revealed that pmoles GlcNAc incorporated into chitin increased with GlcNAc concentration (Fig. 2B, incubations 6–8).

### Average length of *in vitro*-synthesized chitin chains shows dependence on UDP-GlcNAc concentration

**Fig. 3:**
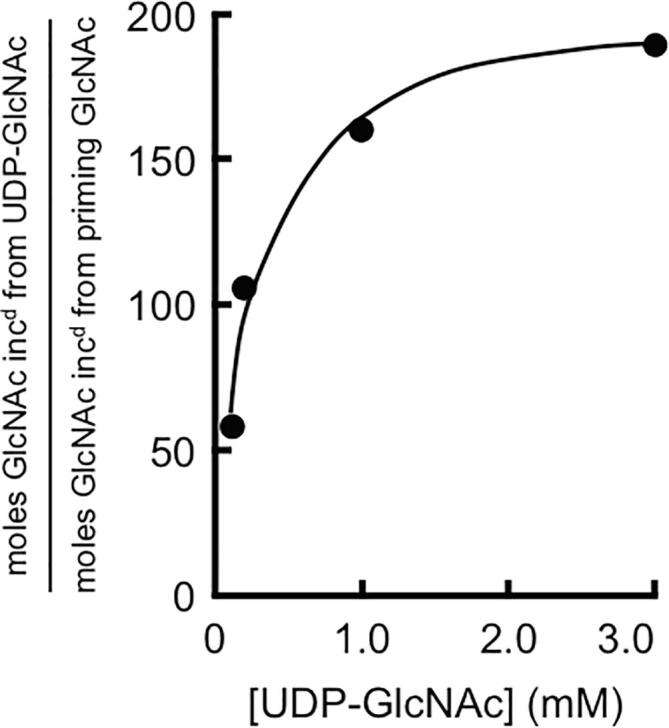
Variation of chitin chain length with UDP-GlcNAc concentration. The ratio of moles of chain-extending, UDP-derived GlcNAc to moles of single priming GlcNAc residues in insoluble chitin was determined for *S. cerevisiae* Chs2 as detailed in Section “Determination of ratios of priming GlcNAc to chain-extending, UDP-derived GlcNAc”. Data points represent the average ratios obtained in three independent experiments (two for one of the data points).

Our finding that incorporation of a single GlcNAc at the end of a chain of insoluble chitin can be quantified presented a way to estimate average chitin chain length by comparing the ratio of priming GlcNAc to chain-extending, UDP-GlcNAc-derived GlcNAc in insoluble chitin. In these experiments, the concentration of GlcNAc was kept constant, but that of UDP-GlcNAc varied, and formation of insoluble chitin by control and Chs2-overexpressing membranes was quantified in two ways. In the first, [^14^C]-labeled GlcNAc and unlabeled UDP-GlcNAc were used as substrates, allowing quantification of priming GlcNAc, and in the second, UDP-[^14^C]GlcNAc and unlabeled GlcNAc were used, allowing quantification of chain-elongating, UDP-GlcNAc-derived GlcNAc. The ratios of moles elongating to moles priming GlcNAc, which give an estimate of average chain length, were plotted against UDP-GlcNAc concentration (Fig. 3). The results show that average chain length is lowest at the lowest UDP-GlcNAc concentration tested (0.1 mM), and that chain length increases as UDP-GlcNAc concentration is raised above Chs2′s reported *K*_m_ for UDP-GlcNAc of 0.8–0.9 mM (Sburlati and Cabib, 1986), approaching a maximum of about 190 GlcNAc residues at the highest UDP-GlcNAc concentration used (3.0 mM). The chain length versus UDP-GlcNAc concentration curve therefore recapitulates the *V* versus [S] curve of a Michaelis-Menten enzyme. Further, the average chitin chain length of 190 made by Chs2 at substrate concentrations approaching saturation is comparable to the values obtained for chitin made by yeast Chs1 and Chs3 *in vitro*, and chitin made *in vivo* in *S. cerevisiae* (Kang et al., 1984, Orlean, 1987, Grabinska et al., 2007). In the latter studies, average chain lengths were estimated following reductive labeling of the reducing end, chitinase digestion, chromatographic separation, and calculation of the specific activities of reduced and non-reduced products of enzymatic digestion.

### UDP-GlcNAc_2_ is not a CS substrate

The [^14^C]GlcNAc-priming assay described in Sections “[^14^C]GlcNAc labeling of COs and insoluble chitin: [^14^C]GlcNAc priming assay” and “Free GlcNAc is incorporated into GlcNAc_2_ and insoluble chitin in a UDP-GlcNAc-dependent manner” offered the opportunity to test for another potential chitin chain initiation reaction. The structure-based model for cellulose and chitin synthesis is for non-reducing end extension without the involvement of a lipid-linked intermediate (Morgan et al., 2013, Dorfmueller et al., 2014), but, if UDP-GlcNAc is a CS’s sole substrate, then chain initiation could involve a separate reaction. One hypothetical reaction would be between two UDP-GlcNAcs, in which the 4-OH of one UDP-GlcNAc displaces UDP from the other, generating UDP-GlcNAc_2_. Class I hyaluronate synthases (HASs), also members of GT Family 2, have been shown to form UDP-linked GlcNAc oligosaccharides (Weigel et al., 2015, Blackburn et al., 2018).

**Fig. 4:**
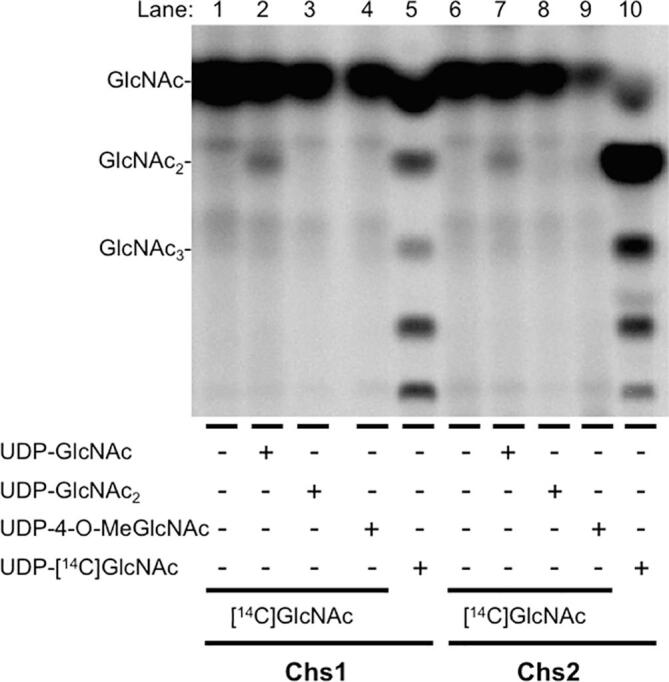
Neither UDP-GlcNAc_2_ nor UDP-4-O-methyl-GlcNAc are substrates for *S. cerevisae* Chs1 or Chs2. [^14^C]GlcNAc priming assays (Section “[^14^C]GlcNAc labeling of COs and insoluble chitin: [^14^C]GlcNAc priming assay”) were carried out on trypsin-treated membranes overexpressing yeast Chs1 (lanes 1–5) or untreated membranes overexpressing Chs2 in the absence of UDP-sugar (lanes 1 and 6) or in the presence of 2 mM concentrations of UDP-GlcNAc (lanes 2 and 7), of UDP-GlcNAc_2_ (lanes 3 and 8), or of UDP-4-O-methyl-GlcNAc (lanes 4 and 9). COs were isolated and separated by TLC. Control incubations with UDP-[^14^C]GlcNAc and 32 mM unlabeled GlcNAc were carried out using the standard CO assay procedure (Section “Standard assay for CO formation”) to verify CO formation by the membrane preparations (lanes 1 and 10).

We used our [^14^C]GlcNAc-priming assay to test whether free GlcNAc, as a proxy for the non-reducing end of a chitin chain, could displace UDP from UDP-GlcNAc_2_ with formation of GlcNAc_3_ or longer COs. For these experiments, we used both overexpressed yeast Chs1 and Chs2. In incubations containing [^14^C]GlcNAc and unlabeled UDP-GlcNAc, [^14^C]GlcNAc_2_ was formed by both CSs (Fig. 4, lanes 2 and 7), whereas no detectable product was formed in the presence of unlabeled UDP-GlcNAc_2_ by either Chs1 or Chs2 (Fig. 4, lanes 3 and 8). We also tested whether UDP-4-O-methyl-GlcNAc was a substrate in this assay, in which case, formation of the disaccharide 4-O-methyl-GlcNAc-GlcNAc would be expected, but no product was formed (Fig. 4, lanes 4 and 9). Therefore, neither UDP-GlcNAc_2_ nor UDP-4-O-methyl-GlcNAc was a substrate in this assay. Further, addition of UDP-GlcNAc_2_ or UDP-4-O-methyl-GlcNAc had no effect on CO formation in incubations containing UDP-[^14^C]GlcNAc and free unlabeled GlcNAc (not shown), indicating that neither compound competes with UDP-GlcNAc. These results therefore provide no support for the notion that UDP-GlcNAc_2_ is a CS substrate, consistent with the findings of Chang and coworkers, who concluded that UDP-GlcNAc_2_ is not a substrate for insoluble chitin synthesis (Chang et al., 2003).

## Discussion

In this study, we tested the predictions that free GlcNAc can prime chitin synthesis *in vitro*, and that at low UDP-GlcNAc concentrations, a CS makes shorter chitin chains. We showed that a fungal CS can indeed carry out a reaction in which free GlcNAc displaces UDP from UDP-GlcNAc, generating GlcNAc_2_, and that the resulting GlcNAc_2_ can be extended into insoluble chitin. This reaction places the priming GlcNAc at the non-reducing end of a chitin chain, and allows the incorporation of priming GlcNAcs at one end of a chitin chain to be quantified. Further, we showed that the average length of *in vitro*-synthesized chitin chains can be estimated from the ratio of moles of chain-extending, UDP-derived GlcNAc to moles of single priming GlcNAc residues in insoluble chitin, and we used this approach to show that the average length of chitin chains increases with UDP-GlcNAc concentration and approaches a maximum at high UDP-GlcNAc concentration. Because the UDP-GlcNAc dependence of CS activity follows Michaelis-Menten kinetics, a CS’s *V*_max_ would be expected to correspond to a maximal rate of monomer addition, hence formation of the longest chains per unit time. We note that the chain length estimated by the new ratio method is dependent on two variables – rate of UDP-GlcNAc concentration-dependent chain extension, and rate of GlcNAc concentration-dependent initiation. The method therefore yields a combined parameter that describes the activities of CSs in terms of relative abilities to initiate on a priming GlcNAc, and then to extend the glycan. The observation that *in vitro* CS activity increases with the concentration of GlcNAc or of 2-acylamido GlcN analogues (Gyore et al., 2014) indicates that the rate of chain initiation and subsequent elongation can be limited by primer availability. Increased rate of priming presents an alternative explanation to allosteric activation for the stimulatory effect of free GlcNAc on *in vitro* CS activity.

The present findings, together with our demonstration that 2-acylamido GlcN analogues are incorporated into COs and stimulate insoluble chitin synthesis (Gyore et al., 2014), are considered next in the context of the model for GlcNAc transfer and transmembrane translocation of the nascent chitin chains (Morgan et al., 2013), and we make new proposals for how CSs can function. For this discussion, it is assumed that CO and chitin synthesis by a yeast chitin synthase in a mixed membrane fraction occur according to the catalysis-translocation model.

### CSs may “self-prime” using UDP-GlcNAc-derived GlcNAc

**Fig. 5:**
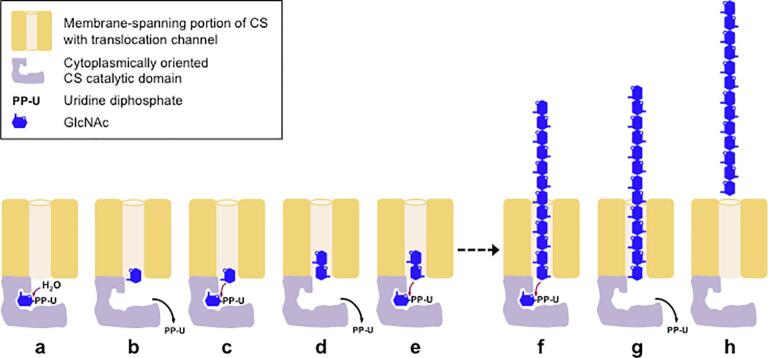
Model for self-priming and synthesis of chitin chains in bursts. CS-catalyzed hydrolysis of UDP-GlcNAc (a) generates a priming GlcNAc (b), which displaces UDP from a second UDP-GlcNAc (c), generating GlcNAc_2_ in the synthase’s translocation channel (d), which in turn displaces UDP from a third UDP-GlcNAc (e). A chitin chain is extended with further transfers of GlcNAc from UDP-GlcNAcs and is extruded through the translocation channel (f). Continued extrusion may remove the non-reducing end of the chitin chain from the active site, with eventual extracellular release of the chain (g and h). Self-priming (a and b) will allow reinitiation of chain synthesis.

The finding that UDP-GlcNAc_2_ is not a CS substrate, and the unlikelihood that UDP-GlcNAc_2_ or a lipid-linked intermediate participate in the CS reaction (Chang et al., 2003, Morgan et al., 2013, Dorfmueller et al., 2014, this study), leave open the question how chitin chains are initiated *de novo*. We show here that free GlcNAc can prime chitin synthesis *in vitro*, a finding that can explain the stimulatory effect of GlcNAc in chitin synthase assays. If GlcNAc is the primer *in vivo*, however, its source is not obvious, because free GlcNAc is not generated during hexosamine biosynthesis (Cabib, 1987, Milewski et al., 2006). One possibility is that CSs have an intrinsic UDP-GlcNAc hydrolyzing activity that allows them to “self-prime” by generating GlcNAc from UDP-GlcNAc, then elongate COs and chitin on that GlcNAc (Fig. 5a–f). In *in vitro* systems, dependence on UDP-GlcNAc hydrolysis is bypassed by free GlcNAc and 2-acylamido GlcN analogues.

If CSs indeed generate GlcNAc from UDP-GlcNAc, then the UDP-GlcNAc hydrolyzing activity of a CS is a potential point of regulation at the level of the synthase itself, for example by post-translational modification of the CS (Lenardon et al., 2010a, Lenardon et al., 2010b, Oh et al., 2012), or interaction with some activator protein (Devrekanli et al., 2012). Furthermore, regulation via self-priming is compatible with the enhanced chitin synthesis that occurs *in vivo* upon cell wall stress and is primarily mediated by upregulation of the UDP-GlcNAc biosynthetic pathway (Ram et al., 1998, Ram et al., 2004, Popolo et al., 2001, Bulik et al., 2003, Levin, 2011, Walker et al., 2010), although additional regulatory mechanisms that render certain CSs less dependent on UDP-GlcNAc concentration may be superimposed on individual CSs *in vivo* (Bulik et al., 2003). It is formally possible that GlcNAc has a dual role as allosteric activator as well as primer of chitin synthesis (Porter and Jaworski, 1966, Camargo et al., 1967, Horsch et al., 1996), but this is hard to reconcile with the fact that 2-acylamido GlcN analogues prime formation of COs that contain one molecule of these analogues, but do not stimulate formation of COs containing only GlcNAc, predicted products if the analogues were allosteric activators (Gyore et al., 2014).

### CSs may synthesize chains in bursts, release them, and then reinitiate

The finding that GlcNAc and 2-acylamido GlcN analogues can displace UDP from UDP-GlcNAc and prime CO and chitin synthesis indicates that these compounds can gain access to the CS’s active site. The non-reducing end of a pre-existing, nascent chitin chain must therefore sometimes no longer be present in the active site. This implies that CSs can release chains from the extracytoplasmic end of the translocation channel, then reinitiate polymerization of a new chain at the catalytic site once a primer such as GlcNAc is present, i.e. that they synthesize chitin chains in bursts (Fig. 5f–h). The possibility that CSs synthesize multiple relatively short chitin chains *in vivo*, rather than continuously extend one long chitin chain, may impact how their products contribute to cell wall construction (see Section “How might different chitin size distributions impact cell wall construction?”).

### A CS’s chitin translocation channel may accommodate side chain modifications on the polysaccharide

The demonstration that 2-acylamido GlcN analogues stimulate insoluble chitin synthesis by Chs2 *in vitro* has implications for the capacity of the chitin translocation channel of CSs. The ability of these analogues to displace UDP from UDP-GlcNAc puts them at the reducing end of a nascent chitin chain, and implies that these residues lead the chains they prime through the translocation channel. The channel must therefore be able to accommodate the bulkier or more polar N-propionyl-, N-butanoyl- and N-glycolyl substituents on the 2-acylamido GlcN derivatives tested (Fig. 1). However, our finding that UDP-4-O-methyl-GlcNAc is neither used as a CS substrate nor competes with UDP-GlcNAc in the CS reaction indicates that the CS active site does not accommodate bulky substituents (in this case CH_3_) at the 4-OH position of GlcNAc in UDP-GlcNAc. It remains to be established whether CSs can use UDP-linked derivatives modified at the 2-position of the sugar, for example, UDP-N-butanoylglucosamine. The possibility of introducing side chain modifications during chitin biosynthesis using UDP-GlcNAc analogs could be exploited to generate polymers with novel properties, or ones bearing groups that could be derivatized post-synthetically.

### How might different chitin size distributions impact cell wall construction?

Phenotypic analyses of chitin synthase-deficient fungal mutants indicate that CSs make different contributions to cell wall structure and biogenesis (Shaw et al., 1991, Roncero, 2002, Ruiz-Herrera et al., 2002, Lenardon et al., 2007, Lenardon et al., 2010a, Lenardon et al., 2010a, Lenardon et al., 2010b, Muszkieta et al., 2014). In part, this is a consequence different patterns of temporal and spatial localization of a fungus’s different CSs (reviewed by Roncero, 2002, Orlean, 2012), but it may also reflect differences between CS products. For example, the *Candida albicans* CS Chs8 generates chitin that forms long microfibrils *in vivo*, whereas the chitin made by *C. albicans* Chs3 forms short rodlets (Lenardon et al., 2007).

Chain length distribution may be “programmed” into a CS. CSs have been assigned to different classes based on amino acid sequence similarities (Roncero, 2002, Ruiz-Herrera et al., 2002), and it is possible that average product chain length made at ambient UDP-GlcNAc concentrations varies in a manner correlated with class membership. Additional factors impacting chain length are UDP-GlcNAc availability, innate UDP-GlcNAc hydrolytic activity – i.e. self-priming capability – and the actions of regulatory proteins. In an example of the latter, prenylation status of the regulatory protein Chs4 impacted length of chitin chains made *in vivo* by *S. cerevisiae* Chs3 (Grabinska et al., 2007).

If chitin chains are synthesized in bursts and extruded into the cell wall reducing end-first as per the glycosyltransfer-translocation model, how are different types of microfibril generated? A key property of chitin, central to its structural role in fungal cell walls, is the ability of individual chains to form interchain hydrogen bonding networks that build microfibrils. Microfibril assembly *in vivo* may be dependent on how CSs are organized in membranes or vesicular structures such as chitosomes (Bartnicki-Garcia, 2006) in which the synthases may operate in multimeric complexes (Sacristan et al., 2013, Gohlke et al., 2017). Were the CSs in a complex to function and be oriented in the membrane according to the structure-based catalysis-translocation model (Morgan et al., 2013), the nascent chitin strands extruded by two or more CSs could readily form parallel interchain hydrogen bonding networks as the chains emerge reducing end-first from their CSs. However, this may not occur *in vivo*, because the crystalline chitin in fungal cell walls contains antiparallel stretches of chains, which are presumed to arise when individual chitin chains fold back on themselves (Lenardon et al., 2007, and references therein). It can be speculated that the type of microfibrillar chitin made by a given CS reflects the size distribution of the chitin it synthesizes. Although it is not clear whether crystalline chitins can be assembled from many relatively short chains, product length may influence the likelihood of chains doubling back on themselves or reorienting relative to one another, and so impact the formation antiparallel hydrogen bonding networks, hence crystal packing and morphology. Consistent with this possibility, a truncated *Rhizopus oryzae* Chs1 variant with a high *K*_m_ for UDP-GlcNAc makes chitin with low crystallinity and was suggested to make short chitin chains (Salgado-Lugo et al., 2016).

In addition to forming structural microfibrils, chitin also contributes to cell wall organization as parts of chitin-β-glucan networks generated by the action of the polysaccharide cross-linkers Crh1 and Crh2. These transglycosylases cleave chitin chains internally, then transfer the newly generated reducing end to a β-glucan acceptor, and can act on nascent chitin chains emerging from a CS, as well as on chitin chains that have been released into the cell wall (reviewed by Arroyo et al., 2016). The extent of Crh1- and Crh2-dependent cross-linking may be impacted the lengths of chitin chains and the frequency at which they are extruded from the synthase.

### Future lines of investigation

The proposals for CS function made here are testable, but will require technical advances. The notion that CSs generate their own priming GlcNAc by UDP-GlcNAc hydrolysis could be explored with a UDP-GlcNAc derivative that is accommodated by the CS active site and releases a diagnostic GlcNAc analogue that cannot itself act as a primer. The possibility of characterizing and comparing CSs in terms of their products requires a high-throughput system to determine average chain length of insoluble chitin, as well as procedures to determine the distribution of chain lengths made by a CS. Such new procedures, and an assay for UDP-GlcNAc hydrolysis, would complement mutational analyses to determine the sequence features of CSs that correlate with rates of chain initiation and extension.

## Declaration of Competing Interest

The authors declare that they have no known competing financial interests or personal relationships that could have appeared to influence the work reported in this paper.

## Footnotes

1 CS, chitin synthase

2 CO, chitin oligosaccharide

3 TLC, thin layer chromatography
